# Narrow Band Imaging with Magnification Can Pick Up Esophageal Squamous Cell Carcinoma More Efficiently Than Lugol Chromoendoscopy in Patients after Chemoradiotherapy

**DOI:** 10.1155/2013/256439

**Published:** 2013-02-12

**Authors:** Itsuko Asada-Hirayama, Shinya Kodashima, Mitsuhiro Fujishiro, Satoshi Ono, Keiko Niimi, Satoshi Mochizuki, Maki Konno-Shimizu, Rie Mikami-Matsuda, Chihiro Minatsuki, Chiemi Nakayama, Yu Takahashi, Nobutake Yamamichi, Kazuhiko Koike

**Affiliations:** ^1^Department of Gastroenterology, Graduate School of Medicine, The University of Tokyo, 7-3-1 Hongo, Bunkyo-ku, Tokyo 113-8655, Japan; ^2^Department of Endoscopy and Endoscopic Surgery, Graduate School of Medicine, The University of Tokyo, 7-3-1 Hongo, Bunkyo-ku, Tokyo 113-8655, Japan; ^3^Center for Epidemiology and Preventive Medicine, The University of Tokyo, 7-3-1 Hongo, Bunkyo-ku, Tokyo 113-8655, Japan

## Abstract

*Aim*. Little is known about the usefulness of narrow band imaging (NBI) for surveillance of patients after chemoradiotherapy for esophageal neoplasia. Its usefulness in detecting esophageal squamous cell carcinoma (SCC) or high-grade intraepithelial neoplasia (HGIN) in these patients was retrospectively compared to Lugol chromoendoscopy. *Patients and Methods*. We assessed the diagnostic ability of NBI with magnification based on the biopsy specimens obtained from iodine-unstained lesions. Seventy-two iodine-unstained lesions were biopsied and consecutively enrolled for this study. The lesions were divided into NBI positive and NBI negative. Sensitivity, specificity, positive predictive value (PPV), negative predictive value (NPV), and accuracy of NBI with magnification and PPV of Lugol chromoendoscopy was calculated using histological assessment as a gold standard. *Results*. Forty-six endoscopic examinations using NBI with magnification followed by Lugol chromoendoscopy were performed to 28 patients. The prevalence of SCC and HGIN was 21.4%. Sensitivity, specificity, PPV, NPV, and accuracy of NBI were 100.0%, 98.5%, 85.7%, 100%, and 98.6%, respectively. On the contrary, PPV of Lugol chromoendoscopy were 8.3%. Compared to Lugol chromoendoscopy, NBI with magnification showed equal sensitivity and significantly higher PPV (*P* < 0.0001). *Conclusion*. NBI with magnification would be able to pick up esophageal neoplasia more efficiently than Lugol chromoendoscopy in patients after chemoradiotherapy.

## 1. Introduction

Posttreatment surveillance of esophageal squamous cell carcinoma (SCC) after chemoradiotherapy (CRT) is very important because early detection of local persistence or recurrence makes it possible to prevent delay in change of treatment. In addition, metachronous SCC of esophagus often develops in the patients with past history of esophageal SCC [1]. However, differential diagnosis between local persistence, recurrence, and metachronous SCC or normal posttherapeutic sequelae such as mucositis and fibrosis often becomes a problem after CRT [2]. Indeed, in our experience, many iodine-unstained areas which were proven to be nonneoplastic lesions by biopsy were frequently observed in patients after CRT for esophageal carcinoma. 

Lugol chromoendoscopy is the gold standard for detecting esophageal SCC [3–6]. Lugol staining is based on a chemical reaction between iodine and the glycogen which is contained in normal epithelial cell microgranules in the stratum spinosum [7]. Dysplastic and cancerous cells are not stained by Lugol's solution because they do not contain glycogen due to their immaturity.

 Recently, narrow band imaging (NBI) (Olympus Medical System Corporation, Tokyo, Japan) is reported to improve detection of esophageal neoplasia in patients at high risk for developing esophageal SCC [2, 8, 9]. It is an optical technique that enhances the diagnostic capability of endoscopes by using narrow-band spectrum optical filters. This filter is designed to correspond to the peak absorption spectrum of hemoglobin to enhance the visualization of mucosal and submucosal microvascular patterns [1]. 

To the best of our knowledge, there are few reports on the feasibility of using NBI endoscopy for the surveillance of esophagus in patients after CRT. In this study, usefulness of NBI with magnification in detecting esophageal SCC or high-grade intraepithelial neoplasia (HGIN) in patients after CRT for esophageal carcinoma was retrospectively assessed in comparison with chromoendoscopy using iodine solution.

## 2. Materials and Methods

### 2.1. Study Subjects

This is a retrospective study. We assessed the diagnostic ability of NBI with magnification based on the biopsy specimens obtained from iodine-unstained lesions. From January 2008 to March 2012, 90 lesions, which were unstained by spraying of Lugol's solution, were biopsied from the patients who had received CRT for esophageal SCC previously and gave written informed consent at the University of Tokyo, Tokyo, Japan; forty-six endoscopic examinations using conventional white light and NBI with magnification followed by Lugol chromoendoscopy were performed to 28 patients. Among the patients who underwent endoscopic examination and biopsy twice or more after CRT, the lesions whichwere judged to have been biopsied previous endoscopic examination on the basis of locations and shapes were excluded from the analysis. Consequently, 72 lesions were consecutively enrolled for this study.

### 2.2. Patient Characteristics

The locations of primary esophageal SCCs were diagnosed by endoscopic examination before CRT. Lymph node metastasis and distant metastasis were diagnosed by computed tomography (CT). The depth of the tumors was generally diagnosed comprehensively based on the findings of endoscopic examination and CT. As an exception, the depth of the tumors was determined based on the pathological assessment in the cases in which CRT was performed as an additional therapy after endoscopic submucosal dissection (ESD).

### 2.3. Endoscopic Procedure

All procedures were performed by using EVIS LUCERA SPECTRUM system (Olympus, Tokyo, Japan) and high-resolution upper gastrointestinal endoscopes, either GIF-Q240Z or GIF-H260Z (Olympus) by 4 endoscopists (S.K., S.O., K.N., and S.M.) with special endoscopic qualification. 

**Figure 1:**
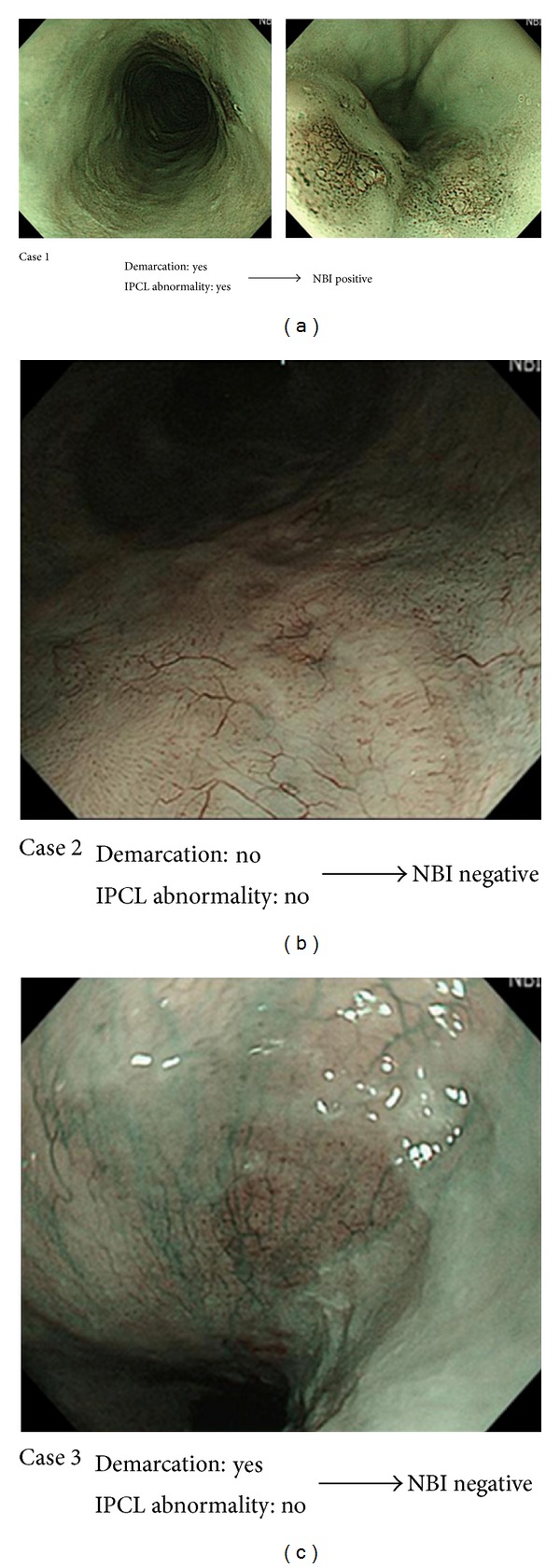
We defined lesions which satisfied both following conditions by NBI with magnification as “NBI-positive”; (1) well-demarcated brownish area, and (2) abnormal changes of intraepithelial papillary capillary loop (IPCL) pattern, that were dilatation, tortuosity, and caliber change in a single IPCL, and variation in the shape of multiple IPCL. We defined lesions which satisfied either or neither of them as “NBI negative.” The abnormal blood vessels observed in Case 2 seemed to reflect neovascularization after chemoradiotherapy, so the lesion was judged to be negative for IPCL abnormality.

**Figure 2:**
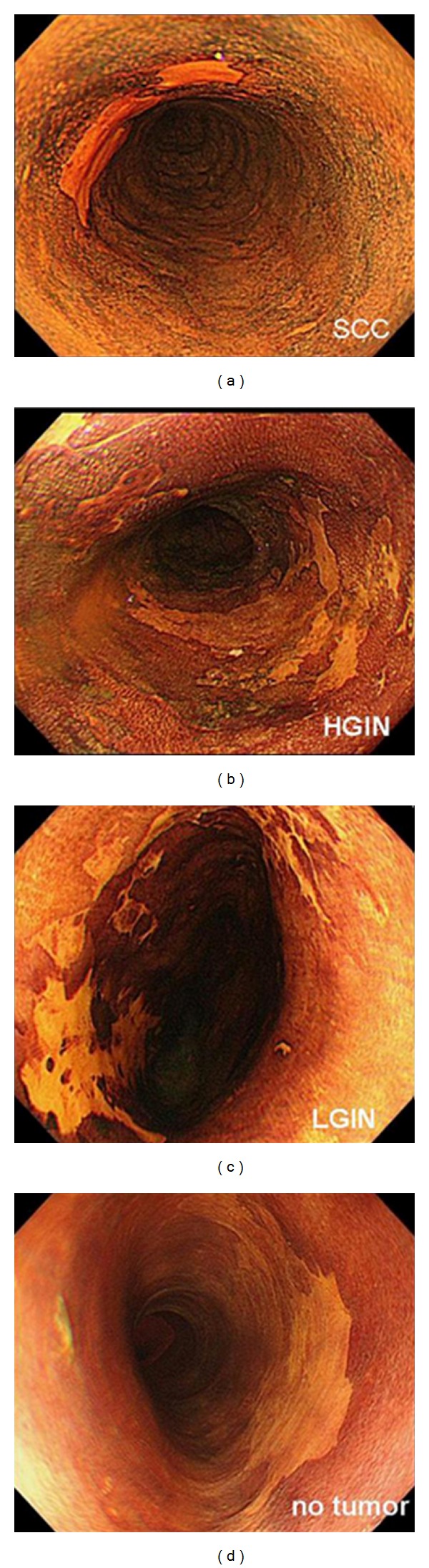
We defined well-demarcated unstained area of more than 5 mm of diameter as “Lugol positive” and performed biopsy. The examples of Lugol-positive lesions were provided here.

 First, the esophageal mucosa was carefully examined by white light and NBI without magnification in order to detect any abnormality of color or surface structure. Second, abnormal lesions found by white light or NBI were examined by NBI with magnification. We defined lesions which satisfied both following conditions by NBI with magnification as “NBI-positive”; (1) well-demarcated brownish area, and (2) abnormal changes of intraepithelial papillary capillary loop (IPCL) pattern, that were dilatation, tortuosity, and caliber change in a single IPCL, and variation in the shape of multiple IPCL [10] (Figure 1). We defined lesions which satisfied either or neither of them as “NBI-negative.” The locations of “NBI-positive” lesions were strictly noted as a distance from the incisor teeth. Third, 10 to 20 mL of 3% Lugol's solution was applied on the esophageal mucosa using a spray catheter passed through the working channel of the endoscope by the same endoscopist who had performed NBI endoscopy. We defined well-demarcated unstained area of more than 5 mm of diameter as “Lugol positive” (Figure 2), and the locations were strictly noted. Lastly, biopsies from “Lugol-positive” lesions were performed, and 2.5% of sodium thiosulfate hydrate was sprayed over the esophageal mucosa to bleach the iodine. 

### 2.4. Pathological Assessment

Biopsy specimens were obtained from each abnormal lesions using forceps. All specimens were soaked in formalin solution and routinely processed. Histological diagnoses were performed by experienced pathologists in our hospital. Histology was assessed according to the following four categories of the World Health Organization (WHO) classification and the Vienna classification [11, 12]: invasive SCC, HGIN, low -grade intraepithelial neoplasia (LGIN), and no tumor. 

### 2.5. Statistical Analysis

Sensitivity, specificity, positive predictive value (PPV), negative predictive value (NPV), and accuracy of NBI with magnification in identifying esophageal SCC and HGIN from the lesions which were Lugol positive were calculated. To clarify how many lesions were SCC or HGIN among Lugol-positive lesions, PPV of Lugol chromoendoscopy was also calculated.

The continuous variables were expressed as medians and ranges. Pearson's *χ* ^2^ test was used to analyze categorical data. *P* value of less than 0.05 was considered significant. Statistical analysis was performed using JMP version 9.0.2 (SAS Institute Japan).

## 3. Results

A total of 46 endoscopic examinations using conventional white light and NBI with magnification followed by Lugol chromoendoscopy were performed to 28 patients. An average number of endoscopic examinations for each patient was 1.6 times. The median age of the 28 patients was 69 years (range: 56–86 years), and the median follow-up duration from CRT to endoscopic examination was 10 months (range: 3–42 months). The location of the primary cancers was shown in Table 1. 

**Figure 3:**
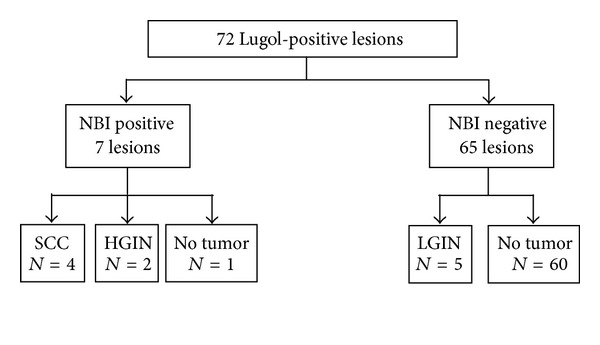
Seventy-two lesions biopsied from the Lugol-positive lesions were analyzed. The lesion observed as a well-demarcated brownish area with abnormal changes of intraepithelial papillary capillary loop (IPCL) pattern by NBI with magnification was defined as NBI positive. SCC squamous cell carcinoma; HGIN high-grade intraepithelial neoplasia; LGIN low-grade intraepithelial neoplasia; *N* = number of lesions.

 A total of 72 lesions biopsied form the Lugol-positive lesions were analyzed. The prevalence of SCC and HGIN was 21.4% (6/28). Seven superficial lesions in 7 patients were diagnosed as NBI positive using NBI endoscopy with magnification before iodine staining. All 7 lesions showed well-demarcated iodine-unstained areas by Lugol chromoendoscopy (Lugol positive). Among them, 4 lesions in 4 patients were histologically confirmed to be SCC, 2 lesions in 2 patients were HGIN, and 1 lesion was no tumor (Figure 3, Table 2). On the other hand, Lugol chromoendoscopy detected 65 iodine-unstained areas which were diagnosed as NBI negative. Among them, 5 lesions in 4 patients were diagnosed as LGIN, and 60 lesions in 20 patients were no tumor (Figure 3). The numbers of lesion detected by NBI with magnification versus Lugol chromoendoscopy were displayed in Table 3.

The sensitivity, specificity, PPV, NPV, and overall accuracy of NBI with magnification for the detection of esophageal SCC or HGIN using histological assessment as the gold standard were 100.0%, 98.5%, 85.7%, 100%, and 98.6%, respectively (Table 4). The PPV of Lugol chromoendoscopy was 8.3% (Table 4). No Lugol-negative lesions were biopsied in this study, so sensitivity, specificity, NPV, and accuracy of Lugol chromoendoscopy were indeterminate. Compared to Lugol chromoendoscopy, NBI with magnification was shown to have equal sensitivity and significantly higher PPV in detecting esophageal SCC or HGIN in patients after CRT (*P* < 0.0001).

## 4. Discussion

In this study, we assessed the diagnostic ability of NBI with magnification in detecting esophageal SCC or HGIN based on the biopsy specimens obtained from the lesions which were unstained by Lugol chromoendoscopy in the patients after CRT. There are many reports describing the superiority of Lugol chromoendoscopy to conventional endoscopy in detection of esophageal neoplasia in high-risk patients [1, 4]. However, the specificity of Lugol chromoendoscopy for the detection of esophageal SCC or HGIN was previously reported to be low, with values ranging from 40 to 95% [3, 4, 13–15]. At the same time, esophageal iodine staining can lead to a transient dysphagia related to an esophagospasm [16], esophagitis [17], and gastritis [18]. Moreover, Lugol chromoendoscopy requires taking some additional time to spray iodine solution and to wait until the whole mucosa is colored.

Recently, NBI is developed as one of the options to achieve optical chromoendoscopy. The benefits of NBI are its visualization of the superficial structure and image enhancement of vasculature within the mucosal layer. In particular, the recognition of changes in the IPCL pattern is useful for the detection of esophageal neoplasia [10]. Many studies reported the usefulness of NBI in detecting esophageal neoplasia in high-risk patients [1, 6, 8]. Furthermore, Chiu et al. reported that NBI with magnification had comparable sensitivity and superior specificity when comparing it to Lugol chromoendoscopy (sensitivity 92.3% versus 92.3%, specificity 91.7% versus 72.2%) [19]. Takenaka et al. also reported that NBI with magnification had comparable sensitivity (90.9% versus 100%, *P* = 1), superior specificity (95.4% versus 84.7%, *P* < 0.001), and superior accuracy (95.1% versus 85.9%, *P* < 0.01), compared with Lugol chromoendoscopy, in detecting esophageal SCC or HGIN in patients with head and neck cancer [20]. 

In this study, we focused on patients after CRT for esophageal carcinoma, among whom differential diagnosis between local persistence, recurrence, and metachronous SCC or normal post-therapeutic sequelae such as mucositis and fibrosis often becomes a problem. As a result, PPV of Lugol chromoendoscopy was only 8.3%. Compared to Lugol chromoendoscopy, NBI with magnification had equal sensitivity and significantly higher PPV (85.7% versus 8.3%) in detecting esophageal SCC or HGIN. This means that NBI with magnification would be able to pick up neoplastic lesions more efficiently than Lugol chromoendoscopy in these patients.

 The prevalence of esophageal SCC and HGIN among the patients after CRT for esophageal carcinoma was 21.4% (6/28) in our study. The importance of regular surveillance for these patients was reconfirmed from this result. A surveillance endoscopy using NBI with magnification may improve acceptability of endoscopic procedures as compared with Lugol chromoendoscopy. Because the use of NBI would avoid all the side effects that can be observed with a Lugol's solution and would shorten the time of endoscopic procedure.

 There were some limitations in this study. First, sensitivity, specificity, NPV, and accuracy of Lugol chromoendoscopy were indeterminate in this study because we assessed the diagnostic ability of NBI with magnification based on the biopsy specimens obtained from the lesions which were unstained by Lugol chromoendoscopy, that means no Lugol-negative lesions were biopsied in this study. However the results of our study suggested that NBI with magnification would be able to pick up esophageal neoplasia in patients after CRT more efficiently than Lugol chromoendoscopy. Second, patients who could not be examined using NBI with magnification because of stricture after CRT were excluded from the analysis because the aim of this study was to compare the diagnostic ability of “NBI with magnification” and “Lugol chromoendoscopy” Third, the diagnostic ability of NBI with magnification for esophageal neoplasia was not compared with that of conventional white light endoscopy. Muto et al. reported that the sensitivity and accuracy of NBI in detecting esophageal neoplasia were significantly higher than that of conventional white light endoscopy based on their multicenter randomized controlled trial [9]. Lastly, it seemed that both NBI with magnification and Lugol chromoendoscopy could not detect submucosal tumorlike SCCs [21]and recurrences [22] which were covered by normal epithelium and grew mainly in the submucosal layer of the esophagus because both methods diagnosed cancerous lesion based mainly on epithelial changes. Therefore other examinations should be considered to detect such lesions. 

## 5. Conclusion

In conclusion, NBI with magnification has an equal sensitivity and significantly higher PPV in detecting esophageal neoplasia in patients after CRT compared to Lugol chromoendoscopy. So it would be able to pick up local persistence, recurrence, and metachronous SCC more efficiently with no side effects related to Lugol's solution. Although a large prospective study is desirable, NBI could replace Lugol chromoendoscopy as a surveillance tool for these patients.

## Figures and Tables

**Table 1:** Baseline characteristics of 28 patients and primary cancers.

Age, median (range), years	69 (56–86)
Sex (male, female)	25, 3
Location	
Ce	1
Ut	3
Mt	15
Lt	9
Ae	0
Depth	
M	1
SM	9
MP or more	18
LN metastasis	
Yes	16
No	12
Distant metastasis	
Yes	5
No	23
Chemotherapy	
CDDP + 5-FU	2
NPD + 5-FU	25
NPD + 5-FU→NPD + S-1	1
Radiation	
Median (range), Gy	50.4 (50–60)
Follow-up period	
Median (range), months	10 (3–42)

**Table 2:** Biopsy results displayed based on NBI with magnification results (number of lesions).

	SCC or HGIN	LGIN or no tumor	Total
NBI-positive	6	1	7
NBI-negative	0	65	65

Total	6	66	72

All 72 lesions were Lugol-positive. SCC: squamous cell carcinoma, HGIN: high-grade intraepithelial neoplasia, LGIN: low-grade intraepithelial neoplasia, NBI: narrow band imaging.

**Table 3:** Number of lesions detected by NBI with magnification versus Lugol chromoendoscopy.

	NBI with magnification	Lugol chromoendoscopy
	*n* (%)	*n* (%)
SCC (4 lesions)	4 (100)	4 (100)
HGIN (2 lesions)	2 (100)	2 (100)
Other (66 lesions)	1 (1.5)	66 (100)

NBI: narrow band imaging, SCC: squamous cell carcinoma, HGIN: high-grade intraepithelial neoplasia.

**Table:** (a)

	Sensitivity	Specificity	PPV	NPV	Accuracy
NBI with magnification	100%	98.5%	85.7%	100%	98.6%

**Table:** (b)

	NBI with magnification	Lugol chromoendoscopy	*P* value
PPV	85.7%	8.3%	*P* < 0.0001

NBI: narrow band imaging, SCC: squamous cell carcinoma, HGIN: high-grade intraepithelial neoplasia, PPV: positive predictive value, NPV: negative predictive value.
